# Treatment of obesity with liraglutide in bipolar disorder: a case report

**DOI:** 10.3389/fpsyt.2026.1855446

**Published:** 2026-07-30

**Authors:** Salvatore Cipolla, Giovanni Vasca, Daniele De Francesco, Sarah Lombardo, Grazia Delle Femine, Antonio Volpicelli, Francesco Perris, Gaia Sampogna, Mario Luciano

**Affiliations:** Department of Psychiatry, University of Campania “Luigi Vanvitelli”, Naples, Italy

**Keywords:** bipolar disorder, case report, GLP-1 receptor agonists, lifestyle, liraglutide, obesity

## Abstract

Bipolar disorder (BD) is a chronic, severe mental illness frequently complicated by metabolic disturbances such as obesity, insulin resistance and dyslipidaemia. We report the case of a 57-year-old man with Bipolar I Disorder and class III obesity (BMI 47.7 Kg/m²) whose mood episodes were tightly linked to weight fluctuations. patients’ psychiatric history began in 1986 with a manic episode and recurrent depressive and manic relapses over nearly four decades, often provoked by weight‐loss attempts. In February 2024, he was admitted for initiation of liraglutide under close psychiatric monitoring, given prior episodes of mania triggered by dietary interventions. At baseline, he exhibited moderate illness severity (CGI = 4), insulin resistance (HOMA-IR = 6.19), dyslipidaemia and sedentary habits. Liraglutide was titrated from 0.6 mg to 1.8 mg over three weeks. During the weight-loss intervention, and temporally after liraglutide titration to 1.8 mg/day, he developed a manic episode (YMRS = 30), prompting an increase in risperidone and addition of gabapentin alongside continuation of mood stabilizers. His manic symptoms remitted within one week, allowing discharge with liraglutide 1.8 mg, risperidone 3 mg, valproic acid and lamotrigine. Over subsequent outpatient follow-up at the target liraglutide dose (3 mg/day), he lost 6.5 kg more (total Δ weight = − 7 kg), his HOMA-IR improved to 5.78 and glycemic and lipid parameters stabilized without further mood destabilization. This case illustrates the bidirectional interplay between metabolic regulation and mood stability in BD, highlights the need for close psychiatric monitoring during structured weight-loss interventions in clinically vulnerable patients, and supports integrated multidisciplinary care when initiating anti-obesity treatments in patients with severe mental illness.

## 1 Introduction

Bipolar disorder (BD) is a chronic and Severe Mental Illness (SMI) characterized by recurrent episodes of mania or hypomania and depression, often interspersed with euthymic phases (1, 2). Affecting approximately 0.53% of the global population (3), BD represents a significant individual and public health burden (4, 5). Beyond the psychiatric symptomatology, BD frequently coexists with substantial psychiatric and medical comorbidities, including social disadvantages, impaired psychosocial functioning, and reduced quality of life (6–9). Among the most concerning medical comorbidities, metabolic disturbances including metabolic syndrome (MetS), insulin resistance (IR), obesity, dyslipidemia, and cardiovascular diseases are the most prevalent (10–12). Approximately 68% of individuals with BD are overweight or obese (13), and up to 50% exhibit IR (14), which is linked to poor response to mood stabilizers and neuroprogression in advanced illness stages (15). BD patients have a 58% higher prevalence of MetS compared to the general population, with increased risks of cardiovascular and cerebrovascular diseases contributing to elevated mortality rates (16).

The underlying pathophysiology of these metabolic disturbances is multifactorial, involving intrinsic disease mechanisms, psychotropic medication side effects, and unhealthy lifestyle behaviors, such as poor diet, physical inactivity, tobacco use, and disrupted circadian rhythms (17–19). Depressive episodes may lead to hyperphagia and inactivity, while manic phases foster impulsive eating and disinhibited behaviors persisting into euthymic phases (20, 21). Smoking rates are notably higher among BD patients, alongside increased substance abuse and lower adherence to healthy dietary patterns like the Mediterranean diet (22, 23). Sedentary behavior and reduced physical activity further compound metabolic risks (15).

Emerging evidence underscores the role of chronic systemic inflammation as a pivotal factor linking BD with metabolic comorbidities (24). Adipose tissue contributes to systemic inflammation by releasing proinflammatory cytokines, with elevated levels of certain inflammatory markers observed in BD patients compared to controls (25). Different mood states in BD (depressive, euthymic, and manic) appear to correlate with distinct cytokine profiles, suggesting dynamic inflammatory shifts throughout the illness course (26, 27). Additionally, BD patients exhibit a heightened risk of obesity with a characteristic inflammatory molecular signature, indicating shared biological pathways (28–31).

Therefore, while metabolic alterations independently pose health risks, they also interact with the course of BD in clinically meaningful ways, and this altered energy regulation (also called “metabolic jet lag”) can be a contributing factor to BD pathophysiology (32). Obesity, MetS, IR, and chronic inflammation may contribute to neurocognitive dysfunction and affective dysregulation, supporting the hypothesis of partially shared biological pathways between metabolic dysfunction and BD (33, 34). Recognizing and addressing these metabolic disturbances are crucial for comprehensive BD management. Pharmacological treatments for BD further complicate the metabolic landscape. Mood stabilizers, including lithium, valproic acid, and carbamazepine, are associated with weight gain (35). Antidepressants can influence body weight, lipid profiles, and glucose metabolism, with some linked to impaired glucose tolerance and type 2 diabetes (36, 37). Second-generation antipsychotics, while effective for mood stabilization, often worsen metabolic parameters, including Body Mass Index (BMI), cholesterol, and glucose levels, particularly with clozapine and olanzapine (38). In recent years, increasing attention has been paid to pharmacological strategies for managing psychotropic drug-related weight gain and obesity in people with SMI. Metformin remains the most established pharmacological option for antipsychotic-induced weight gain, with evidence supporting its use particularly as an early or preventive intervention; however, its effect on established weight gain is generally modest, which may limit its clinical utility in patients with severe obesity or long-standing metabolic burden (39, 40). More recently, GLP-1 receptor agonists have emerged as promising agents for weight reduction in psychiatric populations, including individuals with psychotropic-related weight gain and, in preliminary studies, patients with stable BD and obesity (41–43). Nevertheless, evidence in patients with active or unstable BD remains limited. This gap is clinically relevant because, in BD, body weight, appetite, sleep–wake rhythms, energy expenditure, and mood symptoms may fluctuate together across illness phases (44, 45). Depressive episodes may be associated with hyperphagia, sedentary behavior, weight gain, shame, and reduced self-esteem, whereas manic or hypomanic phases may involve increased activity, reduced sleep, impulsivity, and changes in eating behavior (46). In this context, weight-loss interventions should not be viewed only as metabolic treatments (47). Dietary restriction, increased physical activity, pharmacological appetite modulation, and rapid changes in body weight may also represent psychological and physiological challenges for vulnerable patients. Potential mechanisms include disruption of sleep and social rhythms, changes in reward-related eating behavior, increased salience of body image concerns, and the stress associated with major lifestyle modification (48). However, the specific relationship between intentional weight loss and mood destabilization in BD remains poorly explored.

In this context, psychiatrists play a critical role not only in managing mental and behavioral disturbances but also in supporting the diagnosis and treatment of medical comorbidities through multidisciplinary collaboration. This is especially relevant for metabolic diseases, which may both worsen and be worsened by psychiatric conditions (49–51). Thus, integrating psychiatric and medical care is essential for optimizing outcomes in BD patients (52, 53).

This case report describes a patient with bipolar I disorder and class III obesity, in whom self-esteem and body image concerns emerged as key contributors to psychiatric symptomatology. It aims to illustrate the clinical complexity of managing medically supervised weight loss with liraglutide in a patient with previous mood destabilization during weight-loss attempts.

## 2 Case presentation

M.F. is a 57‐year‐old male with a complex psychiatric and medical history who was admitted to our psychiatric unit in February 2024 for close monitoring during the initiation of an innovative weight‐loss treatment with liraglutide, as weight loss could represent a potential trigger for mood fluctuations. His past medical history is notable for hypothyroidism managed with replacement therapy, a pulmonary hamartoma under periodic surveillance with CT scans, and obstructive sleep apnea syndrome (OSAS) treated with continuous positive airway pressure (C-PAP). A positive family history of psychiatric disorders on the maternal side, a heavy smoking habit (exceeding 20 cigarettes daily), and a sedentary lifestyle further complicate his clinical picture. His substance use history includes cannabis and cocaine abuse between the ages of 18 and 21.

### 2.1 Psychiatric history

M.F.’s psychiatric history dates back to 1986, when at age 19 he experienced a frank manic episode characterized by euphoria and grandiose delusions, which led to a first psychiatric hospitalization lasting 10 days and to a clinical diagnosis of bipolar disorder. During his subsequent evaluation at our unit, the diagnosis was retrospectively confirmed as Bipolar I Disorder according to DSM-5 criteria. Notably, he was already overweight and actively seeking weight loss at that time. Following this initial episode, he developed a depressive phase marked by low mood, apathy, concentration difficulties, hyperphagia, weight gain, and social withdrawal. Despite these symptoms, he managed to complete his studies and establish a long-term relationship, which eventually culminated in marriage. Although the depressive symptoms gradually attenuated, he continued to struggle with low self-esteem and weight gain, ultimately reaching 105 kg at a height of 167 cm.

In 2004, M.F. was admitted involuntarily following a new manic episode featuring inflated self-esteem, prolonged euphoria, an expansive mood, reduced need for sleep, and psychomotor agitation. This episode appeared to be precipitated by family pressure: his wife had urged him to undertake a restrictive diet to improve his overall health. During his second hospitalization, lithium and valproate treatment was initiated for the management of the disorder. Over subsequent years, the patient experienced recurrent mood fluctuations. Depressive phases were typically associated with further weight gain and low self-esteem. Manic phases often emerged after weight-loss attempts and were characterized by heightened self-esteem. These episodes necessitated multiple hospitalizations (six voluntary and one involuntary admissions). From 2021 onward, M.F. was managed on an outpatient basis with monthly follow-ups; however, despite good compliance with his prescribed mood stabilizers, he experienced three further hospitalizations for manic episodes between 2022 and 2023. A chronological overview of the patient’s clinical history is reported in **Table 1**.

**Table 1:** Timeline of the patient’s clinical history.

Year (Age)	Event	Details
1986 (19)	First manic episode	10-day hospitalization; diagnosed with BD Type 1; already overweight and seeking weight loss.
Late 1980s	Depressive phase	Hyperphagia, low mood and concentration; weight rises to 105 kg; social withdrawal.
2004 (37)	Second manic episode	Involuntary psychiatric admission; lithium and valproate initiated.
2004 – 2021	Recurrent mood fluctuations	Six voluntary and one involuntary psychiatric hospitalization; depressive phases characterized by weight gain; manic phases often after weight-loss attempts.
2021 (54)	Transition to our outpatient care Unit	Monthly psychiatric follow-ups; good compliance with mood stabilizers.
2022 – 2023 (55 – 56)	Manic relapses	Three hospitalizations for mania despite treatment adherence.
February 2024 (57)	Admission for liraglutide initiation	Weight 133 kg (BMI 47.7 kg/m²); started liraglutide under close psychiatric and multidisciplinary monitoring.

### 2.2 Current admission and baseline evaluation

Given his distinct mood fluctuation pattern in relation to weight management together with the planned initiation of liraglutide as part of medically supervised weight-loss intervention prescribed by an endocrinologist, the decision was made to admit M.F. for psychiatric monitoring. At admission, his weight was 133 kg, corresponding to a BMI of 47.7 Kg/m2 (consistent with class III obesity). Vital signs were stable: blood pressure was 130/80 mmHg, pulse was 77 beats per minute, and oxygen saturation was 95% on room air.

The endocrinologist prescribed liraglutide starting at 0.6 mg/day subcutaneously for 7 days, with weekly increases of 0.6 mg up to a target dose of 3 mg/day. Concurrently, patients’ psychiatric treatment was adjusted to include risperidone at 1.5 mg/day alongside ongoing mood-stabilizing therapy with valproic acid (1500 mg/day) and lamotrigine (100 mg/day). In addition, the treatment plan incorporated motivational and psychoeducational interventions, a nutritional consultation, and regular weight monitoring.

Baseline laboratory investigations revealed total cholesterol of 205 mg/dL, HDL of 36 mg/dL, LDL of 132 mg/dL, and triglycerides of 185 mg/dL. Fasting blood glucose was 82 mg/dL, with an insulin level of 30.6 μU/mL, yielding a HOMA-IR index of 6.19. The patient’s clinical global impression (CGI) score was 4, indicating moderate illness. His performance on the Personal and Social Performance (PSP) scale suggested only mild impairment in social and daily activities (total score of 76), and the Threshold Assessment Grids (TAG) score was 4. Cognitive assessments, including the Mini-Mental State Examination (MMSE) and additional neuropsychological tests, did not reveal any deterioration. During the mental state examination, M.F. exhibited a hyperthymic temperament with mild irritability. Due to his physical limitations, he relied on assistive devices for certain activities of daily living (e.g., bathing and performing large movements) and was reluctant to accept assistance, expressing significant shame regarding his physical condition. His mobility was notably reduced, yet he remained determined to lose weight and expressed optimism regarding the new pharmacological intervention.

A detailed assessment of his lifestyle prior to admission uncovered a diet predominantly consisting of carbohydrate-rich foods such as pasta, bread, and rice, supplemented by frequent snacking throughout the day. His physical activity was minimal: he commuted via scooter, accumulating less than 30 minutes of walking daily. Additionally, his response on the Seasonal Pattern Assessment Questionnaire (SPAQ) indicated a tendency toward weight gain and low mood during the latter half of the year. The patient’s clinical characteristics at admission are summarized in **Table 2**.

**Table 2:** Patient’s characteristics at admission.

Characteristic	Description
Age & gender	57-year-old male
Diagnosis	Bipolar I Disorder
Comorbidities	Class III obesity; hypothyroidism; lung hamartoma; obstructive sleep apnea syndrome
Clinical features	
Behavioral Domain	Mild psychomotor agitation; reduced mobility due to physical limitations; preserved initiative; determined to engage in weight loss program.
Affective Domain	Hyperthymic mood with mild irritability.
Thought Process and Content Domain	Linear and goal-directed thought process; content focused on somatic concerns and weight reduction; possible mild overvaluation of abilities.
Perceptual Domain	No perceptual disturbances reported or observed.
Sleep	Reduced need for sleep given subthreshold manic features.
Self-care	Partially compromised; uses assistive devices for activities of daily living; expresses shame and reluctance to accept help.
Insight	Partial insight: acknowledges need for treatment and expresses motivation to improve physical health, but limited awareness of potential psychiatric risks.
CGI score	4
PSP global score	76
TAG total score	4

GI, Clinical Global Impression; PSP, Personal and Social Performance scale; TAG, Threshold Assessment Grids.

### 2.3 Hospital course

During the second week of hospitalization, with the liraglutide dosage increased to 1.2 mg/day according to protocol, M.F. demonstrated a notable reduction in irritability and anxiety, although his weight remained stable. However, during the third week of the structured weight-loss intervention, at a time when liraglutide had been titrated to 1.8 mg/day, the patient’s psychiatric condition underwent a marked change. He developed symptoms consistent with a manic episode: incessant, pressured speech, impaired concentration during clinical interviews, a reduced need for sleep, increased goal-directed activity, irritability, and the emergence of grandiose as well as persecutory delusions, notably directed toward his wife. His score on the Young Mania Rating Scale (YMRS) was 30/60, indicating a marked exacerbation of manic symptomatology. In response to this clinical deterioration, psychiatric treatment was promptly adjusted. The risperidone dose was increased to 3 mg/day, the dosage of valproic acid was maintained at 1500 mg/day, and gabapentin at 400 mg/day was added to the regimen. These treatment decisions were guided by current recommendations for the management of acute manic symptoms in BD (54). In particular, the increase in risperidone was consistent with guideline-based recommendations supporting the use of antipsychotics, including risperidone, in acute mania, especially when manic symptoms emerge despite ongoing mood-stabilizing treatment. Valproic acid was maintained as part of the patient’s established mood-stabilizing regimen, given its recognized role in the management of BD and the absence of tolerability issues during hospitalization. Gabapentin was not introduced as a primary antimanic agent, but as an adjunctive symptomatic treatment targeting anxiety and sleep disturbance, in the context of obstructive sleep apnea syndrome, where benzodiazepines were avoided because of potential respiratory risk (55). Over the following week, his manic symptoms progressively stabilized, laboratory parameters returned to normal ranges, and he was deemed suitable for discharge. At the time of discharge, M.F.’s weight had decreased to 132 kg (BMI 47.33). He was scheduled for continued close outpatient monitoring. Upon reaching the target liraglutide dose of 3 mg/day, further improvements were observed: his weight declined to 126.5 kg, the HOMA-IR index improved to 5.78, fasting blood glucose was 88 mg/dL, and insulin levels were 26.6 μU/mL. Importantly, no additional mood fluctuations were documented following these adjustments.

## 3 Discussion

This case report illustrates the complex interplay between metabolic disturbances and psychiatric symptomatology in a patient with Bipolar I Disorder and severe obesity. It also emphasizes the importance of integrated care in individuals with SMI. The management of psychiatric disorders cannot be isolated from the assessment and treatment of physical health comorbidities, especially MetS, which is highly prevalent among patients with BD and significantly affects not only long-term physical outcomes but also response to psychotropic treatment and the course of the psychiatric illness itself.

The patient described herein presented with multiple metabolic abnormalities (obesity, IR, and dyslipidemia) coexisting with a severe and atypical form of BD. The fluctuating weight patterns linked to mood polarity, with depressive phases characterized by hyperphagia and sedentary behavior and manic phases marked by increased activity and decreased food intake, suggest an intrinsic psychopathological-metabolic rhythm that has rarely been systematically described in the literature. The clinical relevance of this case therefore does not reside primarily in the magnitude of weight loss achieved, which was modest, but in the bidirectional metabolic-mood pattern observed in this patient: depressive phases were associated with weight gain and reduced activity, whereas weight-loss attempts were repeatedly followed by manic relapses. In this context, liraglutide should be interpreted as one component of a medically supervised weight-loss intervention that required close psychiatric monitoring, rather than as the primary focus of the report. This distinction is clinically important because the temporal association observed in this case cannot be attributed to liraglutide itself. Rather, the manic relapse occurred within the broader context of a structured weight-loss attempt in a patient with a repeated history of mood destabilization during similar interventions. The case therefore points to a broader vulnerability in which intentional weight loss, regardless of the specific strategy used, may interact with affective instability, body image concerns, reward-related eating behavior, sleep–wake rhythms, and impulse control. While some studies have reported associations between cyclic weight fluctuations and systemic inflammation in BD (45), the distinctive pattern observed in this case further complicates both psychiatric stabilization and the management of metabolic comorbidities.

The introduction of liraglutide in this context warrants particular attention. Liraglutide is a GLP-1 receptor agonist approved for the treatment of obesity and type 2 diabetes (56). It promotes weight loss by delaying gastric emptying, reducing appetite, and improving glycemic control. Preliminary evidence supports its safety and efficacy in individuals with stable BD (41, 42). However, evidence in patients with active or unstable BD remains limited. Therefore, initiating liraglutide in a patient with active psychopathology, treatment resistance, and fragile mood stability requires careful consideration and close monitoring.

In this case, the psychiatric team opted for hospitalization prior to initiating liraglutide therapy because the weight-loss intervention itself was considered clinically sensitive. This decision was not based on the assumption that liraglutide would directly induce manic symptoms, but on the patient’s previous pattern of manic relapses following weight-loss attempts. For this patient, the attempt to lose weight was not a neutral metabolic intervention. It involved a psychologically charged process linked to previous failures, body image concerns, self-esteem, dietary control, behavioral restriction, and the need for sustained motivation. In addition, weight loss interventions often generate emotional strain, require sustained motivation, and impose dietary and behavioral restrictions that can exacerbate affective symptoms in vulnerable patients (57–61). From this perspective, the transient manic relapse observed during the intervention should be interpreted cautiously as occurring in temporal association with the broader weight-loss process, rather than as a direct effect of liraglutide. Hospitalization allowed for the containment of incipient manic symptoms, facilitated a prompt psychopharmacological adjustment, and provided a structured setting in which the patient could begin liraglutide therapy under medical supervision. Although the observed weight loss during hospitalization (−1 kg) was not clinically significant per se, it should be interpreted as a favorable early sign within a longer therapeutic trajectory. Over the following months, the patient lost approximately 7 kg, suggesting that the intervention had a meaningful impact when continued in an outpatient setting. Beyond its role in weight reduction, liraglutide contributed to the improvement of the patient’s lipid profile and insulin sensitivity, which are critical targets in the management of cardiovascular risk in BD. However, in our case, early metabolic improvement occurred alongside a transient manic relapse during the weight-loss intervention, underscoring that metabolic benefit and mood stability may not progress in parallel in clinically vulnerable patients. The structured inpatient setting allowed prompt recognition and management of manic symptoms.

This case underscores the necessity for mental health professionals to acquire a solid understanding of the physical health status of their patients, particularly metabolic functioning, which is often neglected in standard psychiatric assessments. A comprehensive, personalized approach is essential not only for reducing long-term mortality and morbidity but also for improving psychiatric outcomes (23). Recent researches emphasize that the initial management of obesity in patients with SMI should mirror that of the general population, starting with education, diet, physical activity, and behavioral interventions, while also taking into account the side effects of psychiatric medications (62); moreover, with the appropriate support, patients with severe mental disorders can improve their lifestyle and achieve a healthy living (17). When pharmacological treatment for obesity becomes necessary, the choice must be tailored based on the psychiatric profile, risk of drug interactions, and the patient’s ability to adhere to treatment (63).

In this regard, the patient had previously undergone multiple failed attempts at weight loss, including structured dietary programs and admission to a specialized obesity treatment center. These efforts were undermined by a combination of biological and psychological factors, including the metabolic effects of antipsychotic and antidepressant medications (64), emotional dysregulation, and poor impulse control. Notably, in our case, the eventual success of liraglutide in inducing partial weight loss helped to bridge the transition to a more radical intervention (such as bariatric surgery) which was ultimately performed due to persistent severe obesity (BMI 45.36 kg/m²) and comorbidities.

In addition, this case highlights the importance of involving the patient and his family in the treatment process (65). Psychoeducation and motivational strategies aimed at relatives can enhance adherence to lifestyle changes and medication, reduce stigma, and support long-term outcomes (52, 64, 66). In addition, psychotherapeutic interventions, particularly cognitive-behavioral therapy tailored to obesity (CBT-OB), can address the psychological dimensions of disordered eating and weight gain, contributing to a more holistic treatment strategy (67).

### 3.1 Limitations and future directions

This case report is inherently limited by its single-subject design, which prevents generalization. Furthermore, the complex course of the patient’s BD and the simultaneous implementation of multiple interventions (psychopharmacological adjustments, hospitalization, liraglutide therapy) limit the ability to isolate the specific effects of each component. In particular, the improvement of manic symptoms cannot be attributed to a single intervention, and the clinical improvement should be interpreted as the result of an integrated management strategy rather than as the effect of any individual pharmacological adjustment. Moreover, although valproic acid was continued throughout hospitalization, the absence of serum concentration data limits the assessment of mood stabilizer adequacy and should be considered when interpreting both the emergence and subsequent stabilization of manic symptoms. Together with the simultaneous implementation of hospitalization, psychopharmacological adjustments, and liraglutide therapy, this limitation further prevents any attribution of clinical change to a single treatment component. However, the aim of this case report is not to determine the efficacy of a specific treatment component, but to illustrate the clinical complexity of managing weight-loss interventions in a patient with BD, severe obesity, and a documented vulnerability to mood destabilization during weight-loss attempts. Another limitation is the absence of standardized metabolic and psychopathological assessments over time, which would have enabled a more quantitative analysis of outcomes. Future research should focus on controlled studies evaluating the safety and efficacy of GLP-1 receptor agonists in patients with active BD and obesity, ideally including both metabolic and psychiatric endpoints. It would also be valuable to explore predictive factors for successful weight loss in this population, including psychopathological subtypes, treatment adherence, and family involvement.

## 4 Conclusions

This case exemplifies the need for psychiatrists to adopt an integrated biopsychosocial model when managing patients with severe mental illness and comorbid medical conditions. In this patient, previous clinically appropriate weight-loss attempts were repeatedly followed by manic relapses, suggesting a clinically relevant bidirectional relationship between mood stability, body weight, and metabolic health in BD. The initiation of liraglutide within a structured inpatient setting illustrates how psychiatric stabilization and close monitoring may be important prerequisites for anti-obesity interventions in clinically vulnerable patients. Conversely, improved metabolic control contributes to better psychiatric outcomes. The interplay between mind and body is particularly salient in BD, where both pharmacological and behavioral treatments must be tailored to the patient’s fluctuating affective states. Ultimately, interdisciplinary collaboration remains key to optimizing care and improving quality of life for this vulnerable population.

## Data Availability

The original contributions presented in the study are included in the article/supplementary material. Further inquiries can be directed to the corresponding author.
